# Terminal complement pathway activation drives synaptic loss in Alzheimer’s disease models

**DOI:** 10.1186/s40478-022-01404-w

**Published:** 2022-07-06

**Authors:** Sarah M. Carpanini, Megan Torvell, Ryan J. Bevan, Robert A. J. Byrne, Nikoleta Daskoulidou, Takashi Saito, Takaomi C. Saido, Philip R. Taylor, Timothy R. Hughes, Wioleta M. Zelek, B. Paul Morgan

**Affiliations:** 1 UK Dementia Research Institute Cardiff, and Systems Immunity Research Institute, School of Medicine, Cardiff University, Hadyn Ellis Building, Maindy Road, Cardiff, CF24 4HQ UK; 2 Department of Neurocognitive Science, Institute of Brain Science, Nagoya City University Graduate School of Medical Sciences, Nagoya, Japan; 3 Laboratory for Proteolytic Neuroscience, RIKEN Center for Brain Science, Wako, Japan

**Keywords:** Complement, Membrane attack complex, Synapse loss, Alzheimer’s disease

## Abstract

Complement is involved in developmental synaptic pruning and pathological synapse loss in Alzheimer’s disease. It is posited that C1 binding initiates complement activation on synapses; C3 fragments then tag them for microglial phagocytosis. However, the precise mechanisms of complement-mediated synaptic loss remain unclear, and the role of the lytic membrane attack complex (MAC) is unexplored. We here address several knowledge gaps: (i) is complement activated through to MAC at the synapse? (ii) does MAC contribute to synaptic loss? (iii) can MAC inhibition prevent synaptic loss? Novel methods were developed and optimised to quantify C1q, C3 fragments and MAC in total and regional brain homogenates and synaptoneurosomes from WT and *App*^*NL−G−F*^ Alzheimer’s disease model mouse brains at 3, 6, 9 and 12 months of age. The impact on synapse loss of systemic treatment with a MAC blocking antibody and gene knockout of a MAC component was assessed in Alzheimer’s disease model mice. A significant increase in C1q, C3 fragments and MAC was observed in *App*^*NL−G−F*^ mice compared to controls, increasing with age and severity. Administration of anti-C7 antibody to *App*^*NL−G−F*^ mice modulated synapse loss, reflected by the density of dendritic spines in the vicinity of plaques. Constitutive knockout of C6 significantly reduced synapse loss in 3xTg-AD mice. We demonstrate that complement dysregulation occurs in Alzheimer’s disease mice involving the activation (C1q; C3b/iC3b) and terminal (MAC) pathways in brain areas associated with pathology. Inhibition or ablation of MAC formation reduced synapse loss in two Alzheimer’s disease mouse models, demonstrating that MAC formation is a driver of synapse loss. We suggest that MAC directly damages synapses, analogous to neuromuscular junction destruction in myasthenia gravis.

**Supplementary Information:**

The online version contains supplementary material available at 10.1186/s40478-022-01404-w.

## Introduction

Alzheimer’s disease, one of the leading causes of morbidity and mortality in the developed world, is a chronic neurodegenerative disease characterised pathologically by amyloid-β plaques and neurofibrillary tangles made up of hyperphosphorylated tau. Neuroinflammation is a critical driving force in the pathogenesis of Alzheimer’s disease, implicated in multiple aspects of the pathology, including synapse loss, the best known pathological correlate of cognitive decline [1, 2].

Complement, an important component of the innate immune system and defence against bacteria, is a cause of pathological inflammation in many diseases, including brain diseases [3, 4]. Complement dysregulation at sites of pathology attracts and activates pro-inflammatory cells through its chemotactic and anaphylactic products C3a and C5a, and directly damages cells via formation of the membrane attack complex (MAC) pore. Over the last 20 years a substantial body of evidence has accumulated highlighting a critical role for complement in Alzheimer’s disease pathogenesis. Genome wide association studies (GWAS) have identified Alzheimer’s disease risk single nucleotide polymorphisms (SNPs) in complement receptor 1 (*CR1*), clusterin (*CLU*) [5, 6] and *C1S* [7]. Biomarker studies have identified alterations in complement proteins and activation products in blood and/or cerebrospinal fluid (CSF) that distinguish controls, mild cognitive impairment and Alzheimer’s disease patients [8–11]. In post-mortem immunohistochemical studies of Alzheimer’s disease brain C1q, C4b, C3b/iC3b and MAC co-localise with both plaques and tangles [12–15]. Evidence from animal models has proved inconclusive, with complement deficiency protecting from disease in some studies but having no impact, or even an exacerbating effect, in others [16–20].

A role of the complement pathway in the removal of redundant synapses during developmental synaptic pruning was described 15 years ago. In the developing rodent visual system, C1q and C3 fragments localise to and tag synapses for removal [21]. Mice deficient in either C1q, C4 or C3 all showed defects in this developmental synaptic pruning with resultant supernumerary synapses [21–23]. The demonstration that developmental synaptic pruning was also impacted in mice deficient in complement receptor 3 (CR3), the microglial receptor for the C3 opsonic fragment iC3b, provoked the suggestion that synapses destined for elimination were first opsonised with iC3b then engulfed and removed by CR3-expressing microglia [24]. Although these early studies focused on developmental synaptic pruning, the hypothesis that similar complement-mediated processes were responsible for pathological synapse loss in Alzheimer’s disease and other dementias was soon proposed [25, 26]. Subsequent studies in murine models of Alzheimer’s disease supported the hypothesis; pathological synapse loss was reduced by inhibiting or deleting C1q, C3 or CR3, firmly implicating complement activation and opsonisation in synaptic elimination in the disease [18].

These findings underpin the accepted model of how complement causes synaptic loss in Alzheimer’s disease; C1q binds synapses and triggers activation of the classical pathway, coating the synapse with opsonic C3 fragments that signal microglia to bind via CR3, engulf and eliminate. Missing from the current model is any contribution of the MAC, surprising given the evidence that MAC colocalises with Alzheimer’s disease pathology and the well-known roles of MAC in causing cell damage and death in diverse diseases, including brain diseases [15, 27, 28]. Indeed, deficiencies of MAC components and/or MAC inhibition prevent or ameliorate disease in rodent models of stroke, head injury, demyelinating diseases and myasthenia gravis [29–31]. In myasthenia gravis, autoantibodies bind acetylcholine receptors at the neuromuscular junction, a neuron-muscle synapse, then engage C1q to activate the complement classical pathway (CP) and cause MAC-dependent destruction [32]. We hypothesised that MAC played a similar role in the destruction of neuron-neuron synapses in Alzheimer’s disease models. To test this, we first utilised novel assays to measure the presence and distribution of complement activation products in Alzheimer’s model and control mouse brain, and specifically in isolated synaptic fractions. We then tested the effects of systemic administration of a MAC-blocking monoclonal antibody (mAb) or deficiency of the MAC component C6 on synaptic loss in Alzheimer model mice. The data demonstrate that the complement terminal pathway is highly activated in the brain and on synapses in Alzheimer’s disease model mice and that inhibiting or preventing MAC formation reduces synapse loss in the models. The findings change concepts of how synapses are damaged by complement in neurodegeneration and indicate that MAC inhibition may have therapeutic benefit in Alzheimer’s disease and other dementias.

## Materials and methods

### Reagents

All chemicals, except where otherwise stated, were obtained from either Fisher Scientific UK (Loughborough, UK) or Merck (Sigma Aldrich; Gillingham, UK) and were of analytical grade. All plastics were from Invitrogen Life Technologies (Paisley, UK).

### Animal models

All animal procedures were performed in accordance with Animals Scientific Procedures Act 1986 and local institutional guidelines. Animals were group housed in open top cages with 12 h light–dark cycles and food and water available ad libitum. C57BL/6 J (WT; Harlan, Bicester, UK), *C1qa*^*−/−*^, *C6*^*−/−*^ (http://www.informatics.jax.org/reference/J:122430), *C7*^*−/−*^(Jackson ImmunoResearch)*, Cd59a*^*−/−*^, *App*^*NL−G−F*^ and 3xTg-AD mice have been described elsewhere [33–36]. 3xTg-AD mice were backcrossed with *C6*^*−/−*^ mice to generate a 3xTg-AD *C6*^*−/−*^ line. Mice were genotyped for presence or absence of C6 using a customised Taqman SNP assay (AHS09AM). *App*^*NL−G−F*^ knock-in mice carrying the APP Swedish (KM670/671NL), Iberian (I716F), and Arctic (E693G) mutations were obtained from Dr. Takaomi Saido under a material transfer agreement. At appropriate time points, mice were humanely sacrificed with increasing CO_2_ concentration and death confirmed by permanent cessation of circulation. Whole blood was collected by transcardial puncture, left to clot for 10 min at room temperature (RT), incubated on ice for 1 h and centrifuged; resultant serum was stored aliquoted at − 80 °C. Mice were perfused intracardially with phosphate buffered saline (PBS), brains removed, dissected and either fixed or snap frozen, dictated by downstream application. A subset of brains was halved sagitally, one hemisphere immersed in 4% (w/v) paraformaldehyde (PFA) for immunohistochemistry and the other hemisphere snap frozen for western blotting and ELISAs. Another subset of brains were sub-dissected to isolate the hippocampus, frontal cortex and cerebellum; these were immediately snap frozen and stored at − 80 °C. A final subset of brains were allocated for DiOlistic spine labelling either as freshly harvested unfixed hippocampal slices or fixed in 1.5% (w/v) PFA. No significant differences were observed between genders for C1q, C3b/iC3b or terminal complement complex (TCC) in any of the analyses; males and females were used for all ELISA experiments and only male mice for DiOlistics analysis.

### Brain homogenate preparation and synaptoneurosome isolation

Total brain homogenates (TBH) and brain region homogenates were prepared and synaptoneurosomes (SN) isolated based on previously published protocols [37]. In brief, snap frozen brains or brain regions were homogenised in a Dounce glass homogeniser in 1 ml of homogenisation buffer (HB; 5 mM KCl, 1 mM MgCl_2_, 25 mM HEPES, 120 mM NaCl, 2 mM CaCl_2;_ pH 7.5), supplemented with protease inhibitors (cOmplete mini EDTA-free, Roche), and phosphatase inhibitor cocktail V (Millipore). Homogenates were passed through 80 µm nylon filters (Millipore) and either immediately frozen at − 80 °C in 100 µl aliquots as TBH, or passed through a 5 µm filter (Millipore) and centrifuged at 1000xg, 5 min at 4 °C to isolate SN. The pellet from the 5 µm filtrate was washed once by resuspension in HB and centrifugation; washed synaptoneurosome (SN) pellets were frozen at − 80 °C prior to extraction for western blot (WB) and ELISA. SN isolates were validated by WB for the presence of synaptic marker and loss of nuclear marker.

### SDS-page and WB

TBH, SN pellets, isolated hippocampi, cortex and cerebellum were lysed in RIPA buffer (Sigma Aldrich, UK) supplemented with 1 × EDTA-free protease inhibitor (cOmplete mini EDTA-free, Roche) using a handheld motorised homogeniser. Lysed samples were centrifuged at 17,000xg for 20 min at 4 °C and supernatant retained. Total protein concentration was measured using bicinchoninic acid (BCA) kit (Pierce). Samples (25 µg protein) were mixed with 5 × Laemmli sample buffer and resolved on 12.5% (w/v) polyacrylamide tris–glycine electrophoresis gels under non-reducing or reducing conditions. For proteins, gels were stained with Coomassie (0.25% (w/v) Coomassie Brilliant Blue R-250, 40% (v/v) methanol, 10% (v/v) acetic acid). For WB, gels were transferred onto 0.45 µm nitrocellulose membrane (GE Healthcare) and blocked with 5% (w/v) bovine serum albumin (BSA) in PBS containing 0.05% (v/v) Tween-20 (PBS-T) for 1 h at RT. Membranes were incubated with antibodies against PSD95 (Cell signalling technologies, 3450S), Histone H3 (Abcam, ab1791), C1q (Abcam, ab182451) or alpha-tubulin (Abcam, ab7291) or C3 biotinylated overnight in 5% (w/v) BSA at 4 °C. After washing in PBS-T, membranes were incubated in donkey anti-mouse HRP or donkey anti-rabbit HRP secondary antibody (Jackson lmmunoresearch; #715–035-150, #715–035-152) at 1:10,000 or Streptavidin-HRP (R&D Systems; #DY998) at 1 in 1000 for 1 h at RT and washed again in PBS-T. Membranes were developed using enhanced chemiluminescence (ECL, GE Healthcare) and visualised on a SynGene Gbox XX9.

### ELISA for mouse complement proteins and activation products

Mouse C1q was measured in a sandwich ELISA as previously described [38]. In brief, NUNC Maxisorp ELISA plates were coated with anti-C1q mAb (9H10; 5 µg/ml in bicarbonate buffer, pH 9.6), blocked in 2% (w/v) BSA, then sample added; TBH (1 mg/ml), SN (1 mg/ml) or isolated brain region lysates (0.5 mg/ml), all at 50 µl per well, then incubated for 1 h at 37 °C. Mouse C1q purified in house was used as standard. Bound C1q was detected with biotinylated 2F6 anti-C1q (2 µg/ml) for 1 h at 37 °C followed by streptavidin-HRP (1:200, R&D Systems, #890,803) for 1 h at 37 °C. The assay was developed using OPD (SIGMAFAST OPD; Sigma-Aldrich) and absorbance measured at 492 nm (FLUOstar Omega Microplate Reader; BMG LABTECH). TBH isolated from C1q^−/−^ mouse brains was used to confirm assay specificity.

Mouse C3b/iC3b was measured in a novel sandwich ELISA developed for this work. Plates were coated with 2/11 mAb anti-mouse C3b/iC3b/C3c (5 µg/ml, Hycult HM1065) and blocked in BSA (3% w/v)-PBS-T. Standard curves were generated using zymosan-activated mouse serum, batch-generated by activating via both classical and alternative pathways by incubation with Zymosan A (7 mg/ml; Pierce, #21,327) and aggregated human IgG (1 mg/ml; in house) for 32 h at 37 °C in a shaking water bath. The reaction was stopped by centrifugation at 4600 xg for 15 min at 4 °C and the supernatant (activated serum) collected and stored at − 80 °C in aliquots. For standard curves, activated normal mouse serum (Act-NMS) was double-diluted in BSA (1% w/v)-PBST supplemented with 20 mM ethylenediaminetetraacetic acid (EDTA; BSA-PBST-EDTA) from a starting dilution of 1:1000 in duplicate (Additional file 1: Fig. S1a). Samples, including TBH, SN and isolated brain region lysates, were diluted in 1% BSA-PBS-T-EDTA to 7.5 µg/ml-1.0 mg/ml (optimal concentration individually tested for each sample set), added to wells (50 µl in duplicate) and incubated overnight at 4 °C. Bound C3 fragments were detected either with biotinylated rabbit anti-human C3 (in house; cross-reactive with mouse; 1:500 in BSA (1% w/v)-PBS-T; Additional file 1: Fig. S1b) for 2 h at RT followed by streptavidin-HRP (1 in 200 dilution, R&D systems, #890,803) or with HRP labelled rabbit anti-human C3 (1:500). Plates were washed and assays developed with OPD as above.

Mouse terminal complement complex (TCC) was measured in a new ELISA using a novel mouse TCC-specific mAb, 12C3, developed for this work. The assay, like the widely used human TCC assays, measures both the fluid-phase product of terminal pathway activation, sC5b-9, and the membrane-inserted MAC; the term TCC encompasses both complexes. Plates were coated with rabbit anti-rat/mouse C9 (10 µg/ml) [39, 40] and blocked in BSA (3% w/v) -PBS-T. Standard curves were generated starting at 1:3 activated mouse serum then double-diluting in BSA (0.3% w/v) -PBS-T-EDTA (Additional file 1: Fig. S1c). Samples including TBH, SN and isolated brain regions, at 10 µg/ml-2 mg/ml in BSA (0.3% w/v) -PBS-T-EDTA (50 µl per well, duplicates) were incubated for 16 h at 4 °C. Bound TCC was detected by incubation for 1.5 h at RT with anti-TCC mAb 12C3 (5ug/ml in BSA (0.3% w/v) -PBS-T- EDTA) either directly labelled with HRP or unlabelled and followed by HRP conjugated donkey anti-mouse IgG (1:3000 dilution). Plates were washed and developed with OPD. A standard activated mouse serum sample was included as an inter-assay control across all plates and assays to calculate intra- and inter-assay coefficients of variation (%CV; < 10%). Dilution linearity for both ELISAs is shown in (Additional file 1: Fig. S1d).

Mouse CD59a was measured in a sandwich ELISA as above using recombinant mouse CD59a-Fc protein as standard. Plates were coated with mAb 7A6 anti-mouse CD59a (2 µg/ml) and blocked in BSA (3% w/v) -PBS-T. TBH samples (0.5 mg/ml) were incubated overnight at 4 °C. CD59a was detected with rabbit anti mouse CD59a anti-serum (in BSA (0.3% w/v)-PBS-T- EDTA) for 1 h at 37 °C followed by HRP conjugated donkey anti-mouse IgG (1 in 1000 dilution) and developed with OPD. CD59^−/−^ TBH was used to confirm the validity of the assay.

All samples were run in duplicate in all assays. All plates contained inter-assay controls and calibrators.

### Dendritic spine labelling, imaging and analyses

Brains were harvested either unfixed or fixed in situ by perfusion with 1.5% (w/v) PFA. The hippocampus was isolated by dissection from unfixed brains and sliced (200 µm) in a septal to temporal progression using a McIlwain tissue chopper. Time to generate hippocampal slices was less than 10 min from sacrifice, essential for DiOlistic neuronal labelling in unfixed tissue [41]. To eliminate the need for rapid processing, methods were modified to allow DiOlistic labelling in PFA-fixed brains; hemispheres were coronally sliced (80 µm free floating sections) using a Leica VT1200S vibratome (Leica Biosystems); ten slices per mouse containing dorsal hippocampal fields were transferred to superfrost plus histology slides for labelling.

Neuronal DiOlistic labelling was performed as previously described [41–44]. Briefly, 1 µm tungsten particles coated with 1,1′-Dioctadecyl-3,3,3′,3′-Tetramethylindocarbocyanine Perchlorate (DiI; Life Technologies) were fired at a pressure of 80–100 psi onto tissue slices through an inverted cell culture insert (8.0 µm; BD Falcon, BD Biosciences). Labelled, unfixed tissue slices were immersed in Neurobasal-A medium for 20 min at 37 °C with 5% CO_2_ to aid dye diffusion. Labelled fixed tissue slices were immersed in PBS for 1 h at RT to allow for sufficient dye diffusion. Hippocampal neuronal labelling was confirmed by fluorescence microscopy. All slices were post-fixed in 4% (w/v) PFA for 20 min and nuclei stained with Hoechst 33,342 (1:500) in PBS for 10 min. Labelled fixed tissue slices were co-stained for amyloid plaque deposition within the CA1 field using Thioflavin S 0.1% (w/v) (T1892-25G, Sigma) in PBS followed by PBS washes. Slides were subsequently mounted in FluorSave (Millipore).

Slices were confocally imaged with a Leica SP8 Lightning confocal microscope using the 63 × objective (z-axis, interval 0.2 µm) and deconvolved using Leica Lightning Deconvolution software. Secondary dendrites within the striatum radium of CA1 hippocampal dendritic fields were selected based on minimal overlap with adjacent cells; dendritic segments, with the accompanying dendritic spine protrusions were imaged and analysed using Imaris (version 9.2, Bitplane, Zurich, Switzerland). All analyses were performed blind and processed in one batch, in order to eliminate operator bias. Dendritic segments were reconstructed using the Filament Tracer module with default thresholding based around ‘regions of interests’, i.e. dendritic segments typically spanning at least 30 µm. Spine subtype (mushroom, stubby, thin) was automatically defined based on morphology using the SpineClassifer MATLAB extension. In stained Thioflavin S labelled fixed tissue slices, dendritic segments were scored as peri-plaque (< 50 µm from plaques) or distal-plaque (> 50 µm from plaques).

For immunofluorescence quantification of synaptic puncta, brain hemispheres from WT, 3xTg-AD and 3xTg-AD *C6*^*−/−*^ mice were fixed in 4% (w/v) PFA, cryoprotected in 30% (w/v) sucrose, embedded in OCT and cryo-sectioned (10 µm) onto HistoBond slides. Sections were air-dried, washed in PBS, and subjected to citrate-based Heat-Induced Epitope Retrieval and proteinase K Proteolytic-Induced Epitope Retrieval (MC1073930010; Merck). Sections were permeabilised with Triton X 1% (v/v) in PBS for 10 min and blocked with 5% (v/v) normal goat serum in 0.05% (v/v) Tween 20 in PBS for 1 h at RT. Primary antibodies against PSD95 (Abcam ab18258, rabbit) and Bassoon (Synaptic Systems 141,004, guinea pig) were diluted 1:500 in antibody buffer (5% normal goat serum with 0.05% Tween 20 in PBS) and incubated for 48 h at 4 °C. Species-specific Alexa Fluor Plus secondary antibodies (Invitrogen, 1:500) were then applied and incubated for 2 h at RT. Nuclei were stained with DAPI, and endogenous autofluorescence quenched with Sudan Black B. Slides were mounted in FluorSave and stored in the dark at 4 °C until imaging using a Leica SP8 Lightning confocal microscope. For puncta quantification, three Z stack images (Z = 0.6 µm, interval 0.12 µm), consisting of 12 regions of interest (ROI) (excluding nuclei) within the dorsal hippocampal CA1 stratum radiatum field were collected from four mice per genotype. Maximum projections of ROIs were combined into a timeseries and batch processed using the Imaris XTension ‘Normalise Time Points’ to normalise intensities across all images followed by quantification using the Spot function in Imaris.

### Testing whether MAC modulates spine density in AD mice

To test the effects of inhibition of MAC assembly on complement activation and spine density, male *App*^*NL−G−F*^ mice aged 6 months (groups of five) were injected intraperitoneally with the function-blocking anti-C7 mAb 73D1 [39] or D1.3 irrelevant mAb control, each at 40 mg/kg in endotoxin-free PBS, every 3 days for 2 weeks (day 0, 3, 6, 9, 12). Mice were tail bled on days 0 and 3, serum harvested and tested for haemolytic activity in a modified haemolysis assay as described [38, 45, 46]. Mice were sacrificed on day 14, bled and perfused as described above, brains harvested and dissected for quantification of complement activation markers and spine density by DiOlistic spine labelling.

To test the effects of eliminating the capacity to generate MAC, 3xTg-AD mice back-crossed onto *C6* deficiency and unmodified 3xTg-AD mice were compared with WT mice at 12–15 months. Brains were harvested as described, synapse density measured by DiOlistic labelling and C3b/iC3b and TCC quantified in TBH as described above. Excess 73D1 (100 µg/ml) was added to the TCC assay to ensure no interference with the assay (Additional file 1: Supplementary Fig. S1e).

### Statistical analysis

All graphs and statistical analyses were generated using GraphPad Prism 5. The Shapiro–Wilk test was used to check for normal distribution in all datasets. One-way ANOVA with Tukey’s post-hoc test was used to test for differences between age groups of each genotype. Two-way ANOVA with Bonferroni post-hoc test was used to compare between genotypes at each age. Where data were not normally distributed the Mann–Whitney test was used. Unpaired two-tailed t-test was used to compare different groups in the C6 deficiency and anti-C7 treatment experiments and spine densities in DiOlistics; one-way ANOVA with Tukey’s post-hoc test was used to test for differences between each genotype. Results are presented as mean ± standard error of the mean (SEM).

## Results

### Complement activation products are present in brains of ***App***^***NL−G−F***^ mice and enriched at sites of pathology

Brains were isolated from wild type (WT) and *App*^*NL−G−F*^ mice at 3, 6, 9 and 12 months of age. Total brain homogenates (TBH), brain region homogenates and synaptoneurosomes (SN) were prepared as described in methods. Levels of C1q, C3b/iC3b and TCC were measured by ELISA in TBH and SN at each age-point, and region homogenates at 9 months. The specificity of the C1q assay was previously demonstrated [38]. Specificity for the C3b/iC3b and TCC assays was confirmed by absence of signal in C3 deficient and C7 deficient mouse brain homogenates respectively (negative data not shown). All results were expressed relative to total protein measured using the Bicinchoninic Acid (BCA) assay in the samples.

**Fig. 1:**
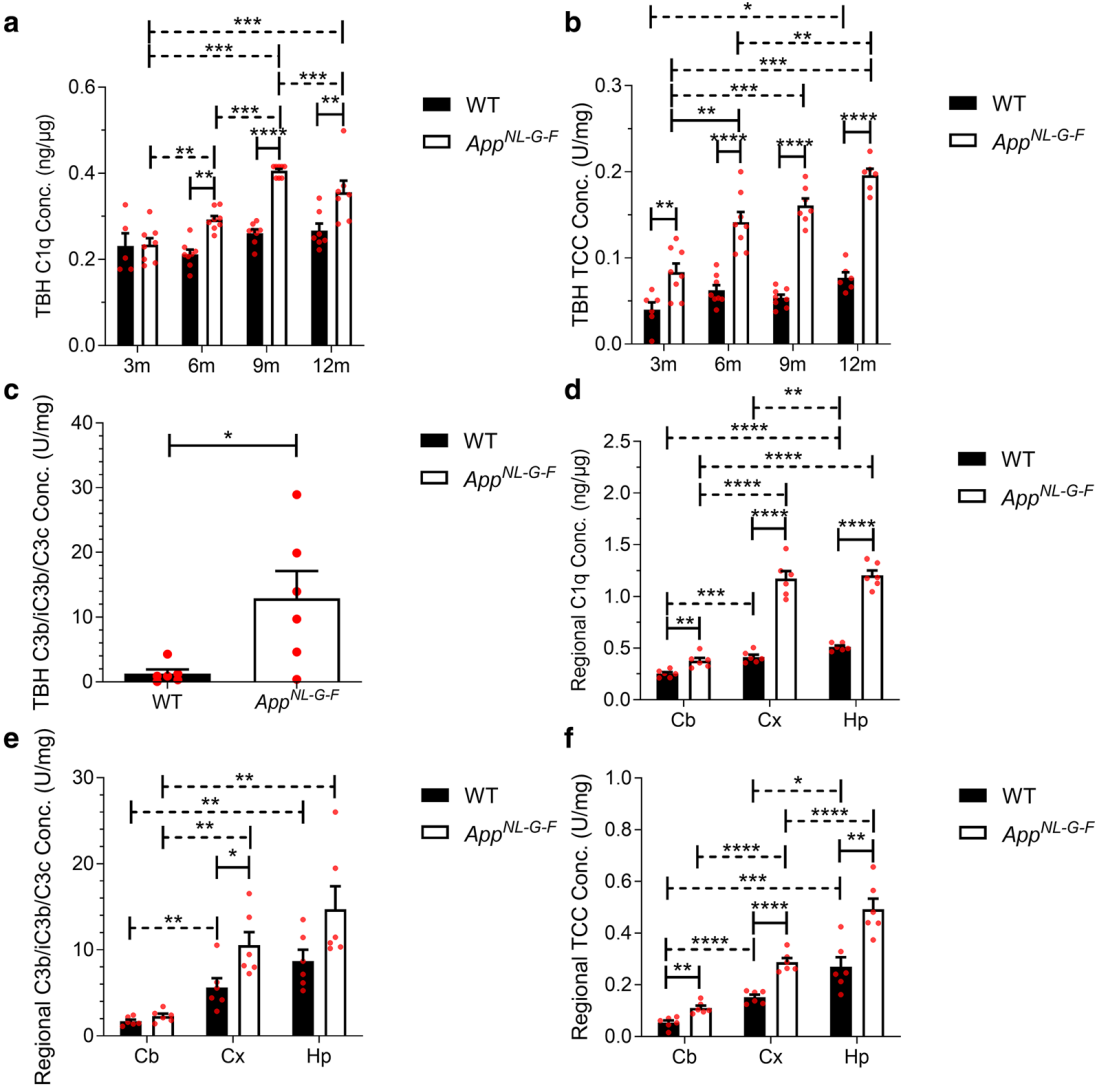
Complement activation products in wildtype and *App*^*NL−G−F*^ mouse brain. **a** C1q levels in total brain homogenate (TBH) were significantly increased in *App*^*NL−G−F*^ at 3, 6, 9 and 12 months (m) compared to age-matched wildtype (WT) mice (*n* = 5–8). C1q levels in TBH remained stable with age in WT mice but increased with age in *App*^*NL−G−F*^ peaking at 9 months of age. One-way ANOVA with Tukey’s post-hoc test was used to test for differences between age groups of each genotype (dashed horizontal lines). Two-way ANOVA with Bonferroni post-hoc test was used to compare between genotypes at each age (solid horizontal lines). **b** Terminal complement complex (TCC) levels in TBH were significantly increased in *App*^*NL−G−F*^ mice at 3, 6, 9 and 12 months (m) compared to age-matched wildtype (WT) mice (*n* = 6–8). TCC levels in TBH showed a small but significant increase with age in WT mice, but were significantly and progressively increased at 6, 9 and 12 months in *App*^*NL−G−F*^ mice. One-way ANOVA with Tukey’s post-hoc test was used to test for differences between age groups of each genotype (dashed horizontal lines). Two-way ANOVA with Bonferroni post-hoc test was used to compare between genotypes at each age (solid horizontal lines). **c** C3 fragment levels in TBH at 6 months of age were significantly increased in *App*^*NL−G−F*^ compared to WT (*n* = *6*). Unpaired two-tailed t-test. **d** C1q levels were highest in hippocampus (Hp), intermediate in cortex (Cx), and lowest in cerebellum (Cb) in WT and *App*^*NL−G−F*^ mice at 9 months. C1q levels were significantly increased in *App*^*NL−G−F*^ compared to age-matched WT in all regions (*n* = *6*). Unpaired two-tailed t-test was used to compare between genotypes (solid horizontal lines) and between regions (dashed horizontal lines). **e** C3 fragments were highest in hippocampus, intermediate in cortex, and lowest in cerebellum in WT and *App*^*NL−G−F*^ mice at 9 months. C3 fragment levels were higher in *App*^*NL−G−F*^ mice compared to WT in all regions, significantly in cortex (*n* = *6*). Mann–Whitney test was used to compare between genotypes (solid horizontal lines) and between regions (dashed horizontal lines). **f** TCC levels were highest in hippocampus, intermediate in cortex, and lowest in cerebellum in WT and *App*^*NL−G−F*^ mice at 9 months. TCC levels were significantly increased in *App*^*NL−G−F*^ compared to WT in all regions tested (*n* = *5–6*). Unpaired two-tailed t-test was used to compare between genotypes (solid horizontal lines) and between regions (dashed horizontal lines). Error bars correspond to SEM. *P < 0.05, **P < 0.01, ***P < 0.001, ****P < *0.0001*

TBH from *App*^*NL−G−F*^ mice contained significantly more C1q at 6, 9 and 12 months compared to age-matched WT (Fig. 1a; solid horizontal lines). While C1q levels remained relatively stable with increasing age in WT mice, levels increased significantly with age in *App*^*NL−G−F*^ (Fig. 1a; dashed horizontal lines). All samples were run in duplicate; the intra-assay % coefficient of variability (%CV) was 2.63%. TCC levels, measured using a novel mouse TCC assay, were significantly elevated in TBH from *App*^*NL−G−F*^ compared to WT mice at 3, 6, 9 and 12 months, threefold higher at 9 and 12 months (Fig. 1b; solid horizontal lines). Notably, TCC levels in WT TBH barely changed with age with no significant difference apart from a borderline significant difference between 3 and 12 months; in contrast, TCC levels in *App*^*NL−G−F*^ TBH markedly increased with age, more than twofold between 3 and 12 months (Fig. 1b; dashed horizontal lines. The TCC ELISA had an intra-assay %CV of 2.75% for TBH. TBH from *App*^*NL−G−F*^ mice contained significantly more C3b/iC3b fragments at 6 months compared to age-matched WT (Fig. 1c).

Levels of C1q, C3b/iC3b and TCC were measured by ELISA in 9 month old WT and *App*^*NL−G−F*^ regional homogenates. The cortex and hippocampus, sites of high amyloid burden in this model, and the cerebellum, an area that is relatively preserved in the model and the human disease, were analysed [47, 48]. C1q levels were significantly elevated in *App*^*NL−G−F*^ compared to WT mice in the three analysed brain regions (Fig. 1d). The cortex and the hippocampus had significantly more C1q than cerebellum (%CV 8.2%). C3b/iC3b levels were elevated in *App*^*NL−G−F*^ compared to WT mice in all three analysed brain regions at 9 months but reached significance only for cortex (Fig. 1e). The C3 fragment ELISA had an intra-assay %CV of 1.43% for isolated brain regions. Measurement of TCC in isolated brain regions showed significantly more TCC in *App*^*NL−G−F*^ mice compared to WT in all brain regions examined (Fig. 1f) (%CV 1.16%).

The large differences in TCC levels between WT and *App*^*NL−G−F*^ TBH prompted measurement of the terminal pathway regulator CD59a to ascertain whether increased TCC levels corresponded with altered levels of CD59a. An ELISA was developed using in-house mAbs and validated using TBH from CD59a^−/−^ mice as the specificity control [34]. CD59a was detected in TBH at 9 months but no significant difference in CD59a levels was observed between WT and *App*^*NL−G−F*^ mice (Additional file 1: Fig. S1f).

### Complement activation products are present at the synapse of ***App***^***NL−G−F***^ mice

SN were isolated from WT and *App*^*NL−G−F*^ mouse brain TBH at 3, 6, 9 and 12 months. Lysed SN pellets were western blotted and probed for PSD95 (post-synaptic marker) to confirm enrichment of synaptic proteins, and histone H3 to demonstrate exclusion of nuclear proteins compared to TBH (Additional file 2: Fig. S2) [49]. Levels of C1q and TCC were measured by ELISA in the isolated SN at the stated ages.

**Fig. 2:**
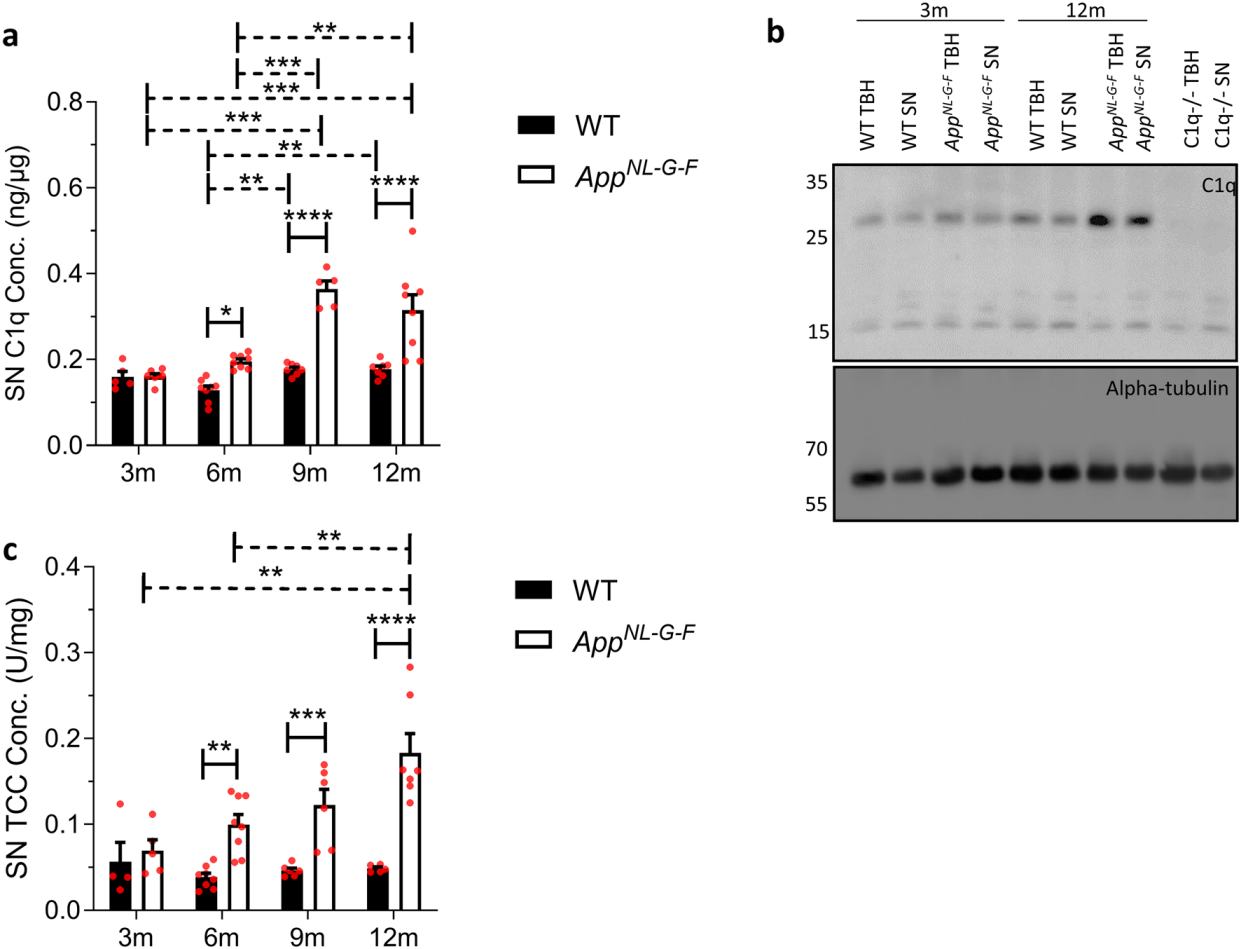
Complement activation products in wildtype and *App*^*NL−G−F*^ mouse synaptoneurosomes (SN). **a** C1q levels were significantly increased in *App*^*NL−G−F*^ synaptoneurosomes (SN) at 6, 9 and 12 months (m) compared to age-matched wildtype (WT) (*n* = *5–8*). SN C1q levels were significantly increased between 6 to 9 months, and 9 to 12 months in WT and between 9 to 12 months in *App*^*NL−G−F*^ SN. One-way ANOVA with Tukey’s post-hoc test was used to test significance of differences between age groups of each genotype (dashed horizontal lines). Two-way ANOVA with Bonferroni post-hoc test was used to compare between genotypes at each age solid horizontal lines. **b** Representative western blot confirming increased C1q levels in TBH and SN in *App*^*NL−G−F*^ compared to WT mice. Alpha-tubulin was used as loading control and C1q-deficient TBH and SN were included as controls. **c** TCC levels were significantly increased in *App*^*NL−G−F*^ SN at 6, 9 and 12 months compared to WT (*n* = *5–8*). TCC levels in SN remained stable with increased age in WT mice but were significantly increased at 9 and 12 months compared with 3 months in *App*^*NL−G−F*^. One-way ANOVA with Tukey’s post-hoc test was used to test for differences between age groups of each genotype (dashed horizontal lines). Two-way ANOVA with Bonferroni post-hoc test was used to compare between genotypes at each age (solid horizontal lines). Error bars correspond to SEM. **P < 0.01, ***P < 0.001, ****P < *0.0001*

Comparison of *App*^*NL−G−F*^ versus WT SN demonstrated significantly increased C1q at 6, 9 and 12 months in *App*^*NL−G−F*^ SN, more than double WT levels at 9 and 12 months (Fig. 2a; solid horizontal lines). C1q levels in WT SN showed a small but significant increase between 6–9 and 6–12 months; in contrast, C1q levels in *App*^*NL−G−F*^ SN were markedly increased at 9 and 12 months compared to 3 or 6 months (Fig. 2a; dashed horizontal lines). Increased C1q in *App*^*NL−G−F*^ TBH compared to WT at 12 months was confirmed in a semi-quantitative manner by WB (Fig. 2b). Comparison of TCC levels in *App*^*NL−G−F*^ versus WT SN demonstrated a highly significant increase in TCC in *APP*^*NL−G−F*^ at 6, 9 and 12 months, more than fourfold greater at 12 months (Fig. 2c; solid horizontal lines). TCC levels in WT SN were low and did not increase with age; however, TCC levels in *App*^*NL−G−F*^ SN were markedly increased, approximately three-fold from 3 to 9 months (Fig. 2c; dashed horizontal lines).

### Systemic C7 inhibition reduces synapse loss in aged Alzheimer’s disease mice

**Fig. 3:**
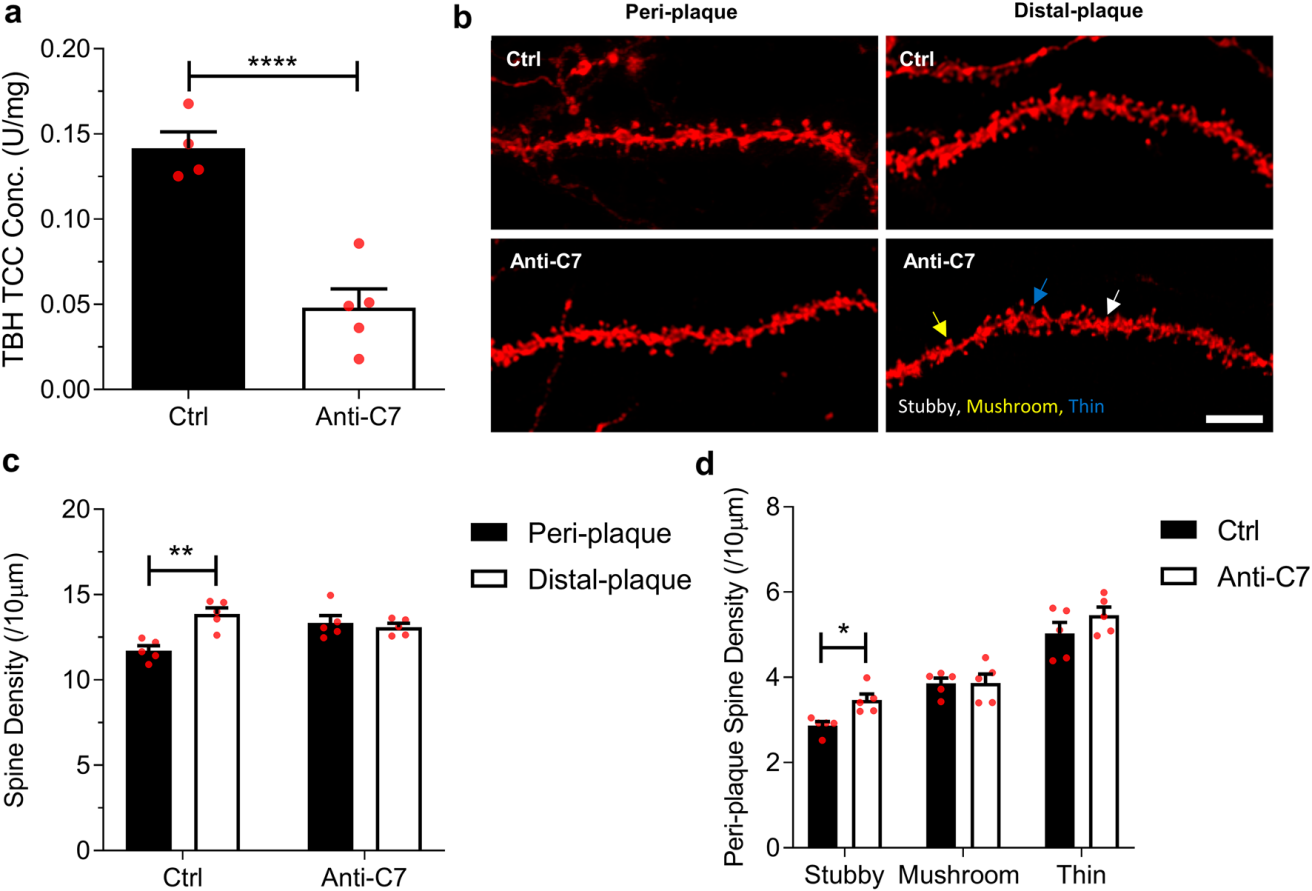
C7 therapeutic inhibits complement and modulates synapse loss in the hippocampus of *App*^*NL−G−F*^ mice. **a** Terminal complement complex (TCC) levels were significantly lower in anti-C7 mAb treated *App*^*NL−G−F*^ mice compared to irrelevant IgG controls at 6 months (*n* = *4–5*). Groups were compared using an unpaired two-tailed t-test. **b** Representative confocal images of DiI labelled CA1 hippocampal dendritic segments from 6 month *App*^*NL−G−F*^ mice treated with anti-C7 mAb or IgG control. Scale bar 5 µm. DiI labelled dendritic spines were analysed from pre-fixed coronal brain slices. Spine densities were analysed from dendritic segments of at least 30 µm. **c** Dendrites were grouped based on proximity to thioflavin-S positive plaques. Dendritic segments within 50 µm of plaques were labelled peri-plaque (see Additional file 3: Fig. S3d), whereas dendrites with no adjacent plaques were labelled distal-plaque. Control IgG-treated but not anti-C7-treated mice showed significant reduction in peri-plaque compared to distal-plaque spine density. **d** Analysis of overall, stubby, mushroom and thin spine density in *App*^*NL−G−F*^ mice treated with control mAb. (C-D, n = 5 mice per group). Unpaired two-tailed t-test was used to compare spine densities between genotypes. Scale bar 5 µm. Error bars correspond to SEM. *P < 0.05, ** P < 0.01, ***P < *0.001*

To explore whether terminal pathway activation impacts neurodegeneration in *App*^*NL−G−F*^ mice, we tested the effects of a systemically delivered terminal pathway-blocking mAb on synapse loss, an early index of neurodegeneration in the model. We first confirmed that, compared to WT, *App*^*NL−G−F*^ mice had significantly reduced synapse numbers in hippocampus at 6 months (Additional file 3: Fig. S3a); overall spine density was significantly reduced and subtype analysis showed that thin spines were most affected (Additional file 3: Fig. S3b). We then treated 6 month old *App*^*NL−G−F*^ mice with the C7-blocking mAb 73D1 [39] for 14 days at a dose and schedule that completely blocked systemic complement activity for the duration of the study (demonstrated in haemolysis assays, data not shown) [39]; control *App*^*NL−G−F*^ mice received an irrelevant control mAb at the same dose and intervals. At day 14, mice were sacrificed, blood collected, intracardiac perfused with PBS to remove complement components in blood vessels, and brains harvested. TCC levels were significantly decreased in TBH from *App*^*NL−G−F*^ mice treated with 73D1 compared to control mAb (Fig. 3a). Directly HRP-labelled 12C3 anti-TCC neoepitope mAb detection antibody was used in these experiments to prevent cross-reactivity with the 73D1 mAb which binds native C7 and C7 in TCC; inclusion of an excess of 73D1 mAb did not impact standard curves in the TCC assay, eliminating the possibility that 73D1 mAb interfered with MAC detection (Additional file 1: Fig. S1d).

Hippocampal dendritic spine analysis was performed using DiOlistics on fixed brain slices, using a modified protocol to include localisation of amyloid plaques (Fig. 3b). Spine numbers and morphological subtypes were quantified in peri-plaque (< 50 µm from plaque) and plaque-distal (> 50 µm from plaque) regions (Additional file 3: Fig. S3c). In control-treated *App*^*NL−G−F*^ mice, spine density was significantly lower in peri-plaque areas compared to plaque-distal as previously reported in other Alzheimer’s disease models [50]; however, in 73D1-treated mice there was no demonstrable significant difference, consistent with a reduction in peri-plaque spine loss (Fig. 3c). Comparison of the different morphological spine subtypes in peri-plaque areas showed increased stubby spine density in the 73D1-treated group compared to controls (Fig. 3d).

### Deficiency of terminal pathway component prevents synapse loss in Alzheimer’s disease mice

**Fig. 4:**
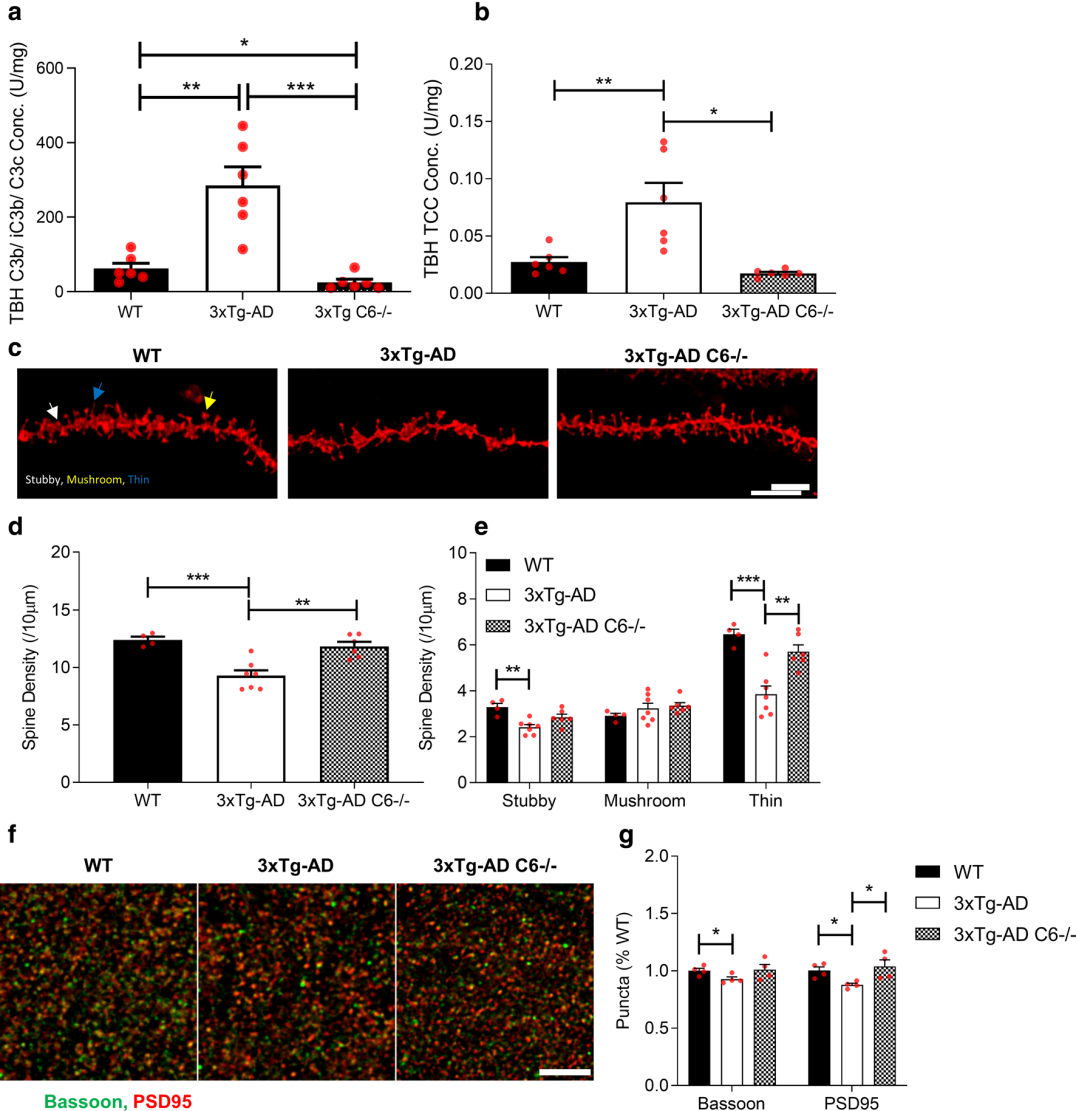
Terminal complement activation is ablated and hippocampal spine loss reduced in C6 deficient 3xTg-AD mice. **a** C3b/iC3b levels were significantly increased in TBH from 12–15 month 3xTg-AD mice compared with matched WT and reduced in 3xTg-AD *C6*^*−/ −*^(*n* = *6*; unpaired two-tailed t-test). **b** TCC levels were significantly increased in TBH from 3xTg-AD compared to WT; TCC levels were markedly reduced in 3xTg-AD *C6*^*−/−*^ mice (all 12–15 months; *n* = *6* per group; unpaired two-tailed t-test). **c** Representative confocal images of DiI labelled CA1 hippocampal dendritic segments from wild type (WT), 3xTg-AD and 3xTg-AD *C6*^*−/−*^*.* Scale bar 5 µm. Spine densities were analysed from dendritic segments of at least 30 µm. DiI labelled dendritic spines were analysed from fresh hippocampal tissue slices and post-fixed following dye diffusion. **d** Spine density was significantly reduced in 3xTg-AD compared to WT; spine density in 3xTg-AD *C6*^*−/−*^ mice was significantly higher than in 3xTg-AD and not different from WT controls. WT, *n* = 4 mice, 3xTg-AD *n* = 7 mice and 3xTg-*AD* *C6*^−/−^ *n* 6 mice. One-way ANOVA with Tukey’s post-hoc test was used to test for differences between each genotype. **e** Sub-analysis of different spine types showed significant reduction in stubby and thin spines in 3xTg-AD mice compared to WT controls and 3xTg-*AD*-*C6*^*−/−*^mice. One-way ANOVA with Tukey’s post-hoc test was used to test for differences between each genotype. **f** Representative images of Bassoon (green) and PSD95 (red) immunoreactive synaptic puncta in the stratum radium of 12-month-old WT, 3xTg-*AD* and 3xTg-*AD* *C6*^*−/−*^ mice. **g** Synaptic puncta, measured by staining with Bassoon or PSD95 and quantified (ROI; 20 µm × 20 µm, twelve per mouse) using Imaris Spot function were reduced in 3xTg-AD mice compared with either WT or 3xTg-AD *C6*^*−/−*^ mice (*n* = 4 per genotype), collated, and analysed. Unpaired two-tailed t-test was used to compare spine densities between genotypes. Scale bar 5 µm. Error bars correspond to SEM. *P < 0.05, **P < 0.01, ***P < 0.001, ****P < *0.0001*

To further explore the contribution of MAC to synapse loss in Alzheimer’s disease model mice we replicated the above analyses in 3xTg-AD mice; we had previously shown that hippocampal dendritic spine loss is a common feature across several AD mouse models, including *App*^*NL−G−F*^ and 3xTg-AD mice [41]. Critically, a C6-deficient 3xTg line had already been established in house. We first sought evidence of early and terminal pathway activation in 3xTg-AD mice by measuring C3b/iC3b and TCC in brain homogenates. Compared to WT controls, C3b/iC3b (Fig. 4a) and TCC (Fig. 4b) levels were markedly increased in TBH from 3xTg-AD mice at 12–15 months. To test whether genetic absence of C6, and hence MAC-forming capacity, impacts hippocampal dendritic loss, we measured hippocampal dendritic spine density in the hippocampus at 12 months in 3xTg-AD and 3xTg-AD *C6*^*−/−*^ mice (Fig. 4c). As previously reported [41], 3xTg-AD mice at this age showed a marked reduction in spine density compared to WT mice; in contrast, the overall spine density in 3xTg-AD *C6*^*−/−*^ was comparable to that in WT mice; comparison of C6-deficient and C6-sufficient 3xTg-AD mice confirmed a highly significant difference in spine density (Fig. 4d). Comparison of the different morphological spine subtypes showed a significant loss of stubby and thin spines in 3xTg-AD mice but not in 3xTg-AD *C6*^*−/−*^ mice (Fig. 4e). Mushroom spines were not significantly lost in 3xTg-AD mice compared to WT and not impacted by C6 deficiency (Fig. 4e). Impact of C6 deficiency on synapse loss in 3xTg-AD was further tested by immunofluorescence staining of synaptic puncta using the pre-synaptic marker Bassoon and post-synaptic marker PSD95 (Fig. 4f). Both pre- and post- synaptic markers were significantly reduced in the 3xTg-AD mice compared to WT; in contrast, 3xTg-AD *C6*^*−/−*^ mice showed puncta densities that were comparable to WT mice and significantly greater than C6-sufficient 3xTg-AD, confirming that prevention of MAC assembly protects against complement-driven synaptic loss (Fig. 4g).

## Discussion

There is an abundance of evidence demonstrating complement activation in the Alzheimer’s disease brain but only limited understanding of whether and how complement contributes to Alzheimer’s disease pathology. Evidence includes the co-localisation of complement proteins and activation products with amyloid plaques and tau tangles in post-mortem Alzheimer’s disease brain [12–15], and the presence of complement activation products in Alzheimer’s disease CSF and plasma [8–11]. Evidence from animal models of Alzheimer’s disease has implicated complement as a trigger for and driver of synaptic loss, an early and critical event in neurodegeneration that strongly correlates with cognitive decline; indeed, mice deficient in C1q, C4 or C3 all show defects in developmental synaptic pruning leading to accumulation of supernumerary synapses [51].

**Fig. 5:**
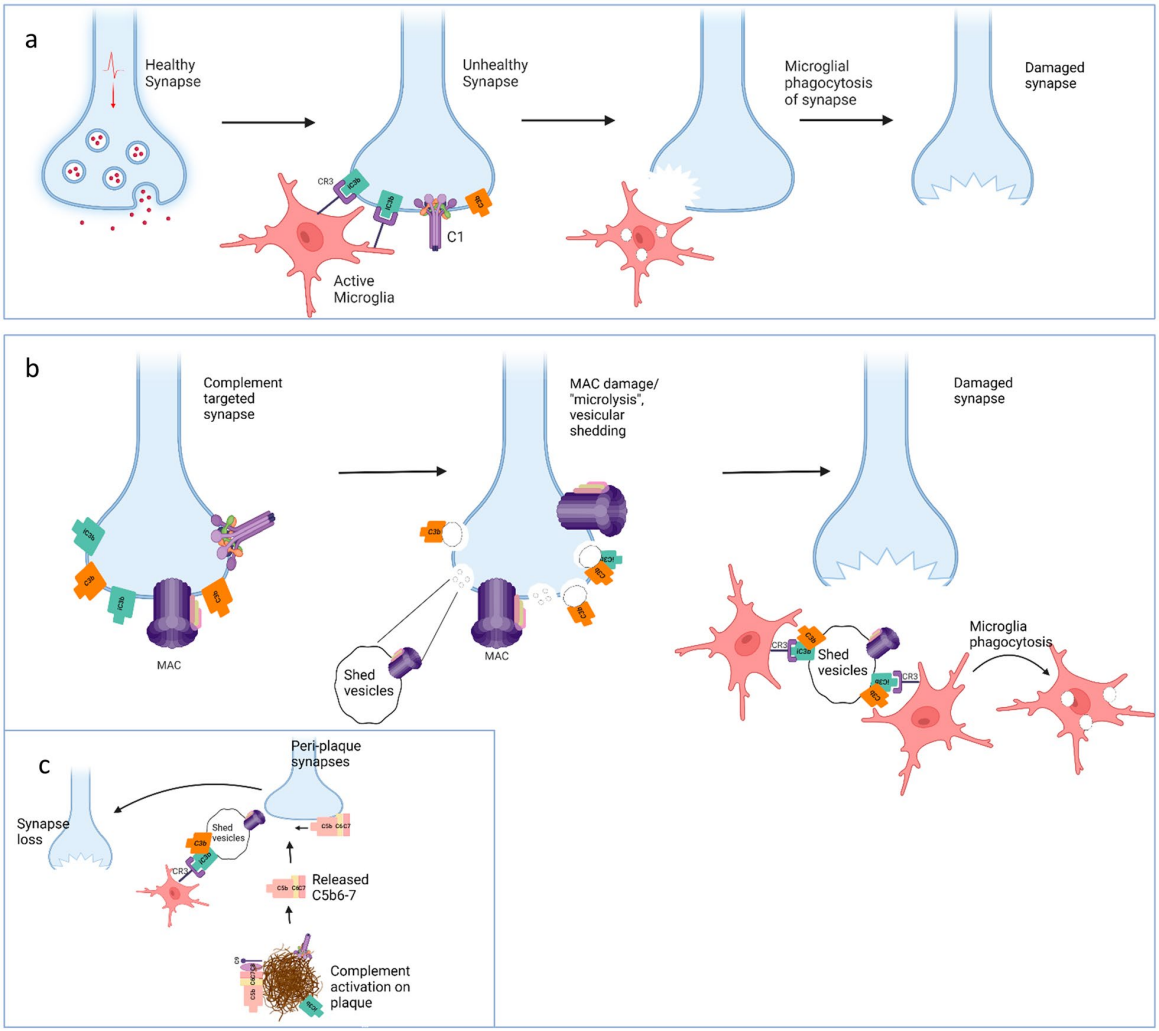
Proposed role of membrane attack complex (MAC) in complement mediated synapse loss. **a** In the current model for synapse elimination, C1q in the C1 complex binds an unknown tag on synapses for removal. Activated C1 then cleaves C4 and C2 to generate the C3 convertase C4b2a. The convertase cleaves C3 to C3b which binds the synapse membrane. C3b is cleaved to iC3b by factor I in the presence of cofactors. iC3b-tagged synapses are then engulfed by microglia expressing the iC3b receptor CR3. **b** In the revised model, complement is activated on synapses as above but proceeds through to formation of MAC, resulting in shedding of complement-opsonised synaptic fragments and “microlytic” destruction of the synapse. Microglia then phagocytose the opsonised fragments. **c** Peri-plaque synapses may be subject to bystander seeding of MAC precursors (C5b-7) as a result of plaque complement activation, leading to MAC formation on the synapse with consequences as in (**b**). Figure created with Biorender.com

The first step in complement-driven synaptic loss involves binding of C1, the initiator of classical pathway activation, to synapses destined for elimination. This involves engagement of the synapse by C1 via C1q, the pattern recognition moiety of the classical complement pathway. Precisely what C1q recognises on the synapse remains unclear, although numerous “flags”, including pentraxins and apoptotic markers, notably phosphatidyl serine exposure, have been suggested [52–54]. As in other contexts, conformational changes in surface-bound C1 induce activation of its enzymatic component C1s that sequentially cleaves C4 and C2 to generate the surface-bound C3 convertase C4b2a. Cleavage of C3 by the convertase generates C3b which covalently binds the synapse membrane. The enzyme factor I, in the presence of appropriate cofactors, cleaves C3b, leaving iC3b on the synapse surface. In the current model for synapse elimination, iC3b-tagged synapses are then engulfed by microglia expressing the iC3b receptor CR3 (CD11b/CD18) (Fig. 5a) [21–23]. The mechanism by which microglia remove opsonised synapses from the neuronal processes is unclear; in other contexts, microglial phagocytosis involves the engulfment of debris and dead cells appropriately tagged for recognition [55]. It has been suggested that microglia might “chew off” the synapses, a process termed trogocytosis, important in lymphocyte biology but with limited evidence for microglia and none that implicates complement [56].

Generalised myasthenia gravis is a complement-mediated disease involving destruction of the neuromuscular junction (NMJ), a synapse linking the motor neuron with the muscle cell [32]. Involvement of MAC in the process was demonstrated more than 40 years ago [57]; complement activation leads to MAC formation at the NMJ that causes “microlysis” and shedding of NMJ membranes coated with complement activation products. Infiltrating microglia then eliminate this debris through phagocytosis. The essential role of MAC in NMJ destruction was subsequently confirmed in rodent models of generalised myasthenia gravis where C6 deficiency prevented NMJ destruction and disease [31, 58], and in man where MAC-inhibiting drugs block NMJ destruction and ameliorate disease; indeed, C5-blocking drugs are now in the clinic for therapy of myasthenia gravis [31, 32]. Despite this powerful precedent, there has been no effort to ascertain whether MAC forms on brain synapses resulting in damage that may precede or precipitate synaptic loss. Here we present evidence that complement mediated synaptic loss in neurodegeneration is indeed MAC-dependent.

We first sought evidence that complement dysregulation in Alzheimer’s disease models involved activation through the terminal pathway with production of MAC. We confirmed that levels of C1q and C3 activation fragments were increased in the models as anticipated from previous studies [18, 59, 60]. We then used a novel assay that measures both MAC and the soluble sC5b-9 complex, generically termed TCC. In two mouse models, *App*^*NL−G−F*^ and 3xTg-AD, TCC levels in brain were markedly elevated in older mice compared to matched WT controls, demonstrating that the terminal pathway was activated. In *App*^*NL−G−F*^ mice, brain areas reported to have the heaviest amyloid burden in the model contained the highest amounts of TCC; further, the age-related increase in C3b/iC3b and TCC levels correlated with the reported acquisition of AD pathology in the *App*^*NL−G−F*^ model [47]. Isolated synaptoneurosomes from these mice, positive for the anticipated complement markers C1q and C3b/iC3b, were also strongly positive for TCC, providing the first direct demonstration of TCC/MAC deposition at the synapse in an Alzheimer’s disease model. Although the source of complement proteins in the normal and pathological brain remains to be determined, expression of terminal pathway components has been reported in control and AD brain post-mortem [61] and in normal mouse brain [62].

Presence of MAC at the synapse does not alone provide evidence that it is contributing to synaptic loss. In order to address the functional relevance of MAC at the synapse in Alzheimer’s disease models, we first investigated the effect of a terminal pathway-blocking anti-C7 mAb on synapse loss in the *App*^*NL−G−F*^ model. We chose this model because we have previously demonstrated significant synapse loss at 6 months [41], the age selected for anti-C7 treatment. Here we replicated these findings and showed that loss of thin spines predominated. The antibody was administered intraperitoneally and completely blocked systemic complement activity over the 14-day course of the experiment. Remarkably, the systemically administered antibody significantly reduced TCC levels in brains of treated mice at day 14, suggesting that enough antibody to block terminal pathway activation accessed the brain over the treatment period. BBB impairment in the context of mild inflammation has been reported in the *App*^*NL−G−F*^ model [63]. We measured spine density and morphology, robust and highly sensitive parameters that provide a reliable index of synaptic dysfunction [64]. Overall spine density was not significantly different between anti-C7 and irrelevant antibody control treated mice at day 14, provoking us to compare plaque-distal and peri-plaque areas; the latter are most affected in Alzheimer’s disease models and man and peri-plaque synaptic loss is the earliest pathological indicator of neurodegeneration [50, 65]. Spine density was significantly lower in peri-plaque compared to plaque-distal areas in control *App*^*NL−G−F*^ mice, but this difference was lost in anti-C7 treated *App*^*NL−G−F*^ mice, implying an effect of C7 inhibition on spine loss. Peri-plaque stubby spine density was significantly higher in anti-C7-treated versus control *App*^*NL−G−F*^ mice.

Although the observed effects of systemic anti-C7 therapy on spine density were subtle, we considered it remarkable that even a brief period of MAC inhibition with a systemically administered antibody impacted spine loss. To further explore and validate the finding we utilised a second Alzheimer’s disease model, the 3xTgAD mouse, already back-crossed to C6 deficiency in-house. We first demonstrated elevated TCC levels in brain extracts from 3xTgAD mice compared to matched controls, confirming that the terminal pathway was activated in this model. We then used DiOlistics to test whether C6 deficiency, which prevents MAC formation, impacted dendritic spine loss in 3xTgAD mice. As expected from our previous work [41], 3xTg mice at 12 months showed a significant reduction in spine density compared to age-matched WT controls. Spine morphology analysis showed that thin spines, the most plastic and dynamic subtype, were most impacted in the 3xTg-AD model, similar to our observations in *App*^*NL−G−F*^ mice. Selective reduction in thin spine density has also been reported in other neurodegeneration models and in the aged mouse, provoking the suggestion that they are the most susceptible spine type in neurodegeneration [66, 67]. In 3xTg-AD mice, C6 deficiency completely prevented the loss; spine density in 3xTg-AD *C6*^*−/−*^ mice was not different from controls and was markedly increased compared to complement-sufficient 3xTg-AD mice in both DiOistic and immunohistochemical analysis.

These data conclusively show that, in Alzheimer’s disease models, as in generalised myasthenia gravis, terminal pathway activation and MAC formation are required for pathological synapse loss and suggest that thin spines may be particularly vulnerable. We suggest a revised model for complement-mediated synapse loss in which complement activation on the targeted synapse proceeds through to MAC formation causing “microlytic” shedding of complement-opsonised synaptic fragments and eventual destruction of the synapse (Fig. 5b). The proposed model and the previous model are not mutually exclusive. Although we show a requirement for MAC in pathological synapse loss, microglial contribute through clearance of iC3b-opsonised synaptic debris and may also facilitate the elimination of MAC-damaged synapses. Importantly, levels of C3b/iC3b were also lower in C6-deficient AD model mouse brain, indicating that early pathway activation and opsonisation was reduced in the absence of MAC-mediated damage, likely impacting microglial engagement of opsonised synapses and synaptic fragments. The selective loss of peri-plaque synapses observed in models and man suggests an additional mechanism whereby complement activation on plaques initiates bystander injury of adjacent synapses through seeding of MAC precursors (Fig. 5c); a precedent has been reported in neuromyelitis optica, where aquaporin-4 autoantibodies trigger complement activation on astrocytes, releasing MAC precursors that seed damaging MAC assembly on nearby neurons [68].

MAC-induced membrane shedding is a well-described process in many cell types, acting to protect the cell from lytic killing; however, non-lytic MAC is not without consequence, allowing ion flux into the cell and initiating multiple pathways, including activation of the inflammasome and other pro-inflammatory systems, and apoptotic triggers [69, 70]. Whether MAC formation at the synapse initiates such events locally or globally in the neuron remains to be ascertained. Future work should be directed at understanding the impact of MAC at the synapse on neuronal health and exploring whether complement bystander injury through seeding of the MAC precursor complex C5b67 occurs on peri-plaque synapses in Alzheimer’s disease.

Implication of MAC in pathological synapse loss opens the prospect of new therapies for neurodegenerative diseases. Numerous drugs targeting the terminal pathway, mostly at the C5 stage, are already in the clinic, including for generalised myasthenia gravis and neuromyelitis optica [71, 72]. The evidence of impact of systemic therapy in this study and the recent suggestion that the blood brain barrier is impaired in Alzheimer’s disease, particularly around plaques, might provoke consideration of whether current therapeutics can access the brain [73–77]. Novel brain-penetrant MAC-blocking drugs, developed either through modification of existing drugs or creation of new ones, could provide a much-needed new approach to therapy of neurodegenerative diseases [78].

## Supplementary Information

**Additional file 1**: **Fig. S1.** Generation and characterisation of novel ELISAs to measure C3 fragments (C3b/iC3b) and TCC (a) C3b/iC3b ELISA showing clear differentiation between non-activated vs activated (with zymosan) mouse serum. (b) WB of mouse plasma under reducing and non-reducing conditions to demonstrate that the rabbit anti-human polyclonal C3 antibody used in the assay recognises mouse C3. (c) TCC ELISA showing clear differentiation between non-activated versus activated (with zymosan) mouse serum. (d) Dilution linearity for C3b/iC3b and TCC ELISAs. (e) Addition of excess 73D1 mAb had no effect on TCC standard curves. (f) CD59 levels in TBH at 9 months of age showed no significant difference between genotypes (n=5-6).

**Additional file 2**: **Fig. S2.** Validation of synaptic protein isolates (synaptoneurosomes) (a) Representative western blot from two wildtype (WT) and two *App*^*NL-G-F*^ mice at 12 months of age showing enrichment of synaptic marker (PSD95) and loss of nuclear marker (histone H3) in synaptoneurosome (SN) compared to total brain homogenate (TBH). (b) Representative Coomassie stained total protein gel to demonstrate equal protein loading and no protein degradation in the preparations.

**Additional file 3**: **Fig. S3.** Hippocampal spine loss in *App*^*NL-G-F*^ mice Spine density analysis of overall (a) and stubby, mushroom and thin (b) spine density in 6 month old wildtype and AppNL-G-F mice. Error bars correspond to SEM. Unpaired two-tailed t-test was used to compare spine densities between genotypes (WT n=4 mice, AppNL-G-F n=8 mice). * P<0.05, ** P<0.01, *** P<0.001, **** P<0.0001. (c) Representative picture to illustrate peri-plaque synapse loss; plaque is stained with Thioflavin S (blue) and dendritic spines with DiI (red). Note the absence of proximal spines protrusions closer to the plaque. Scale bar is 5µm.

## Data Availability

Reagents are available upon reasonable request to the corresponding author.
