# Supplementary figures and images for: Terminal complement pathway activation drives synaptic loss in Alzheimer’s disease models
Source: Acta Neuropathol Commun. 2022 Jul 6;10:99. doi: 10.1186/s40478-022-01404-w (PMC9258209; doi:10.1186/s40478-022-01404-w)

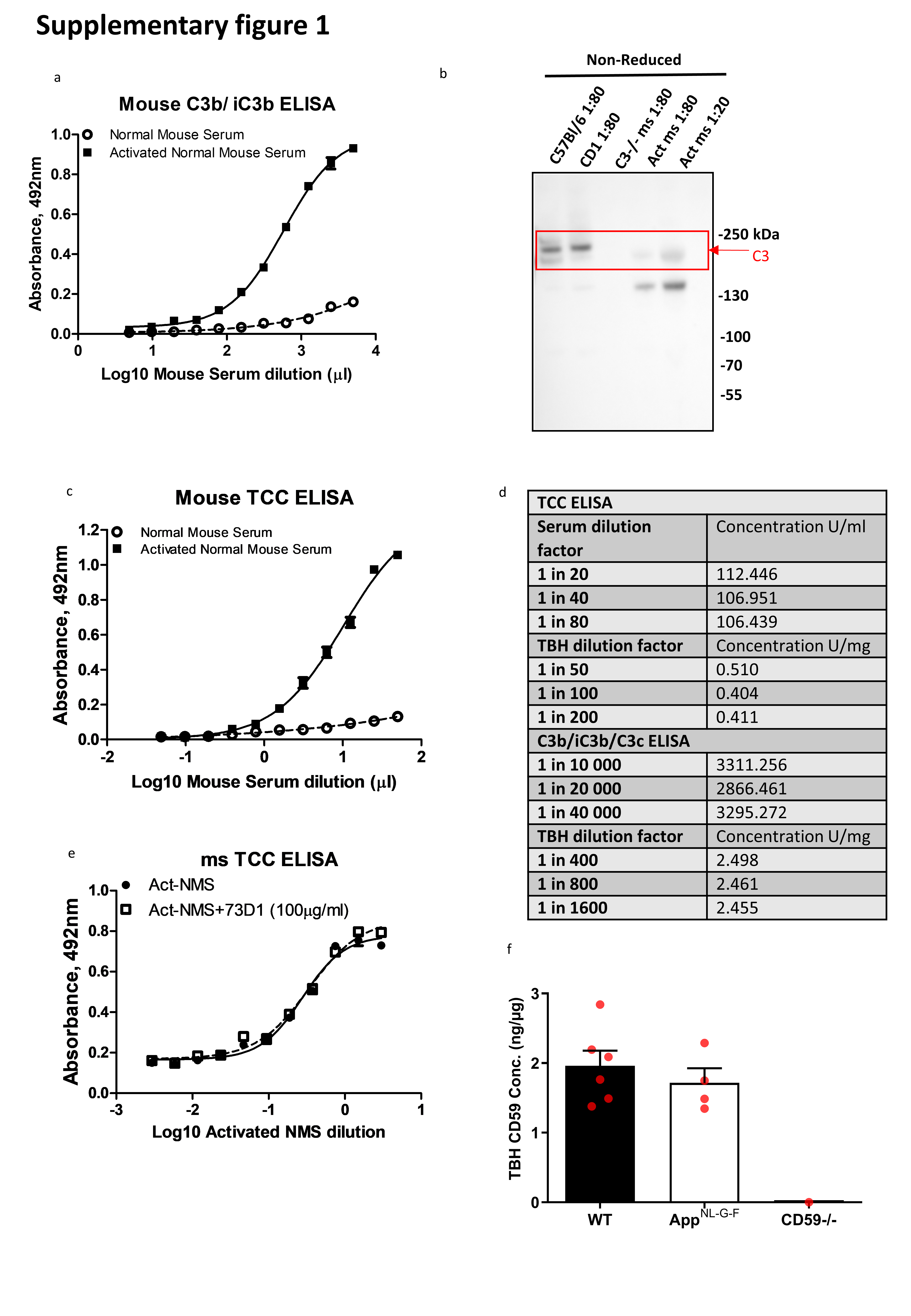

Supplement: Supplementary file 1 — Additional file 1: Fig. S1. Generation and characterisation of novel ELISAs to measure C3 fragments (C3b/iC3b) and TCC (a) C3b/iC3b ELISA showing clear differentiation between non-activated vs activated (with zymosan) mouse serum. (b) WB of mouse plasma under reducing and non-reducing conditions to demonstrate that the rabbit anti-human polyclonal C3 antibody used in the assay recognises mouse C3. (c) TCC ELISA showing clear differentiation between non-activated versus activated (with zymosan) mouse serum. (d) Dilution linearity for C3b/iC3b and TCC ELISAs. (e) Addition of excess 73D1 mAb had no effect on TCC standard curves. (f) CD59 levels in TBH at 9 months of age showed no significant difference between genotypes (n=5-6). [file 40478_2022_1404_MOESM1_ESM.tif]

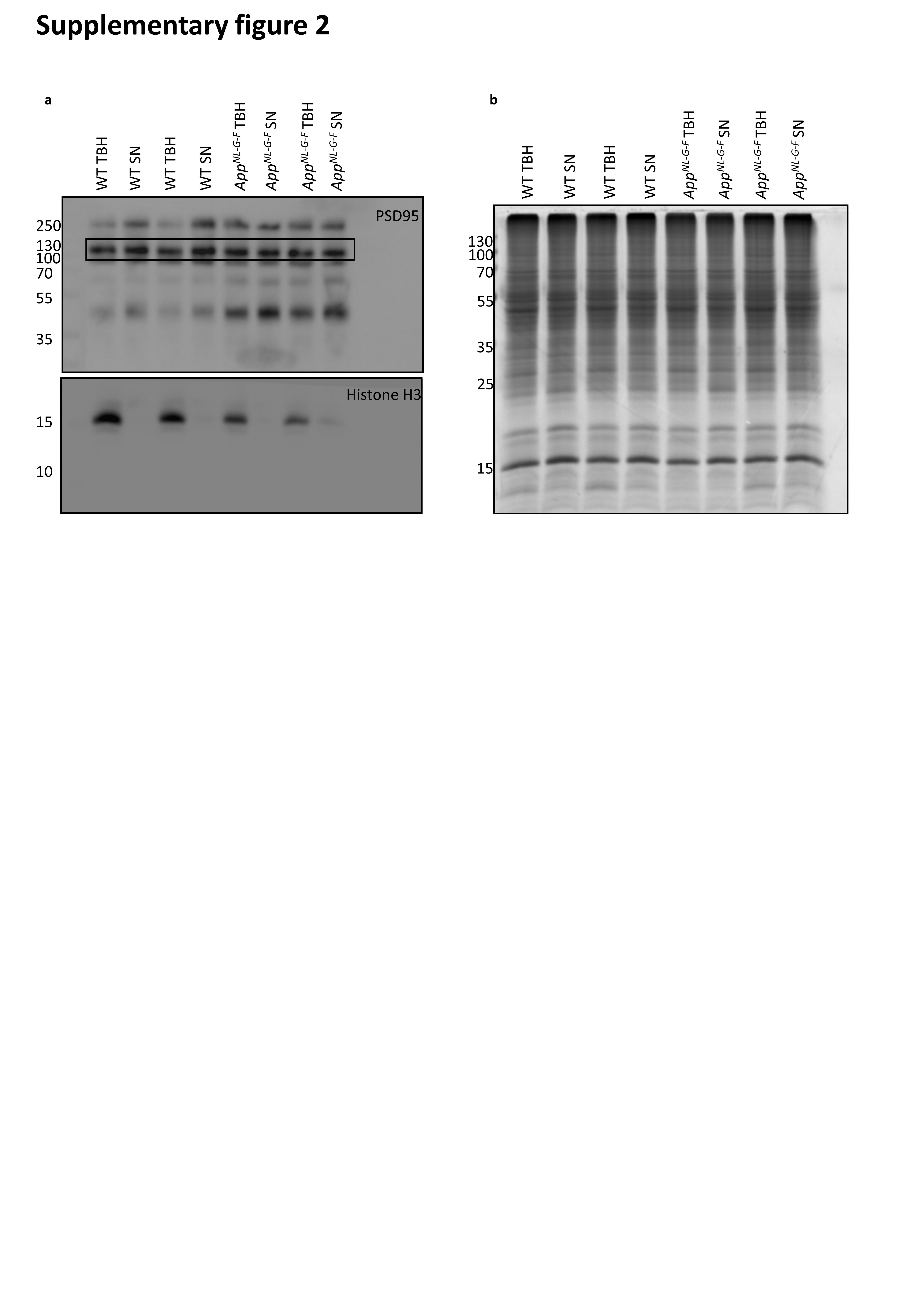

Supplement: Supplementary file 2 — Additional file 2: Fig. S2. Validation of synaptic protein isolates (synaptoneurosomes) (a) Representative western blot from two wildtype (WT) and two AppNL-G-F mice at 12 months of age showing enrichment of synaptic marker (PSD95) and loss of nuclear marker (histone H3) in synaptoneurosome (SN) compared to total brain homogenate (TBH). (b) Representative Coomassie stained total protein gel to demonstrate equal protein loading and no protein degradation in the preparations. [file 40478_2022_1404_MOESM2_ESM.tif]

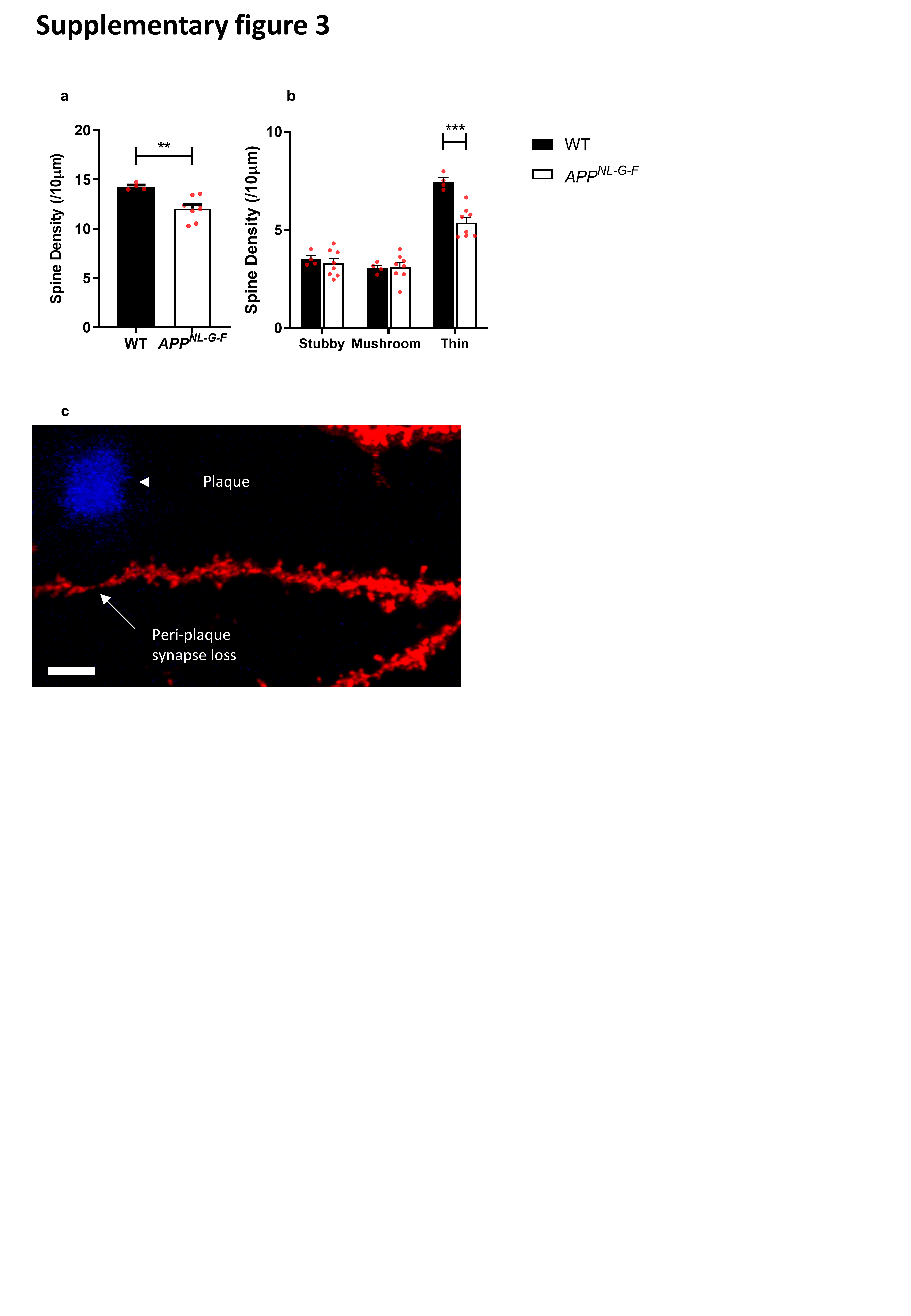

Supplement: Supplementary file 3 — Additional file 3: Fig. S3. Hippocampal spine loss in AppNL-G-F mice Spine density analysis of overall (a) and stubby, mushroom and thin (b) spine density in 6 month old wildtype and AppNL-G-F mice. Error bars correspond to SEM. Unpaired two-tailed t-test was used to compare spine densities between genotypes (WT n=4 mice, AppNL-G-F n=8 mice). * P<0.05, ** P<0.01, *** P<0.001, **** P<0.0001. (c) Representative picture to illustrate peri-plaque synapse loss; plaque is stained with Thioflavin S (blue) and dendritic spines with DiI (red). Note the absence of proximal spines protrusions closer to the plaque. Scale bar is 5µm. [file 40478_2022_1404_MOESM3_ESM.tif]
